# ‘Struggling to participate in everyday life’: emerging adults’ experiences of living with long-term health challenges

**DOI:** 10.1186/s12889-023-16291-6

**Published:** 2023-07-17

**Authors:** Anurajee Rasalingam, Idunn Brekke, Una Stenberg, Mette Haaland-Øverby, Sølvi Helseth

**Affiliations:** 1 Faculty of Health Sciences, Department of Nursing and Health Promotion, Oslo Metropolitan University, Oslo, Norway; 2 Frambu Resource Centre for Rare Disorders, Siggerud, Norway; 3 National advisory unit on learning and mastery in health, Oslo university hospital, Oslo, Norway

**Keywords:** Participation, Disability, Emerging adults, Long-term health challenges, Quality of life, Young adults

## Abstract

**Aim:**

To gain a deeper understanding of the experiences of participation in the everyday life of emerging adults living with long-term health challenges and how this influences their own quality of life.

**Methods:**

Using an explorative study design, data were collected through in-depth interviews with a sample of 12 young people aged 18–29 years living with long-term health challenges in Norway.

**Findings:**

The analysis identified one overarching theme of struggling to participate in everyday life, and four subthemes: the notion of being independent but also dependent, experiencing mismatch between needs and support, experiencing deprivation of spontaneity and feeling uncertain about the future. The emerging adults experienced difficulties with participation in key areas of life such as education, employment and leisure activities. Associated symptoms of their diagnosis, limited physical abilities and lack of sufficient support made it hard to participate in everyday life the way they aspired to.

**Conclusion:**

The challenges of living with a long-term health challenge as an emerging adult contributed to limitations in participation in different areas of life that was perceived as important for their quality of life.

**Supplementary Information:**

The online version contains supplementary material available at 10.1186/s12889-023-16291-6.

## Introduction

Engagement in the economic, social and cultural life of a community is crucial for the health and quality of life of young people [1]. Medical and technological development has led to an increasing number of adolescents with long-term health challenges (somatic health condition and physical impairment) surviving into adulthood [2]. Many long-term health challenges, depending on the severity, have complex consequences with a significant impact on the individual’s everyday life, influencing their opportunities to participate in the community [3], and young people living with long-term health challenges may experience disabilities. This paper draws on a biopsychosocial understanding of disability in line with the World Health Organization (WHO). In their International Classification of Functioning, Disability and Health (ICF), disability is referred to as the negative aspects of the interaction between individuals with a health condition and personal and environmental factors [4]. In the ICF, participation is defined as ‘involvement in life situations’ [5], which can be described in different ways such as “taking part” and “engaged in an area of life” in everyday life. According to Hammel et al. [6] participation is influenced by environmental factors at the individual level (such as immediate support from family and friends), community level (such as social networking, community groups and capital), and societal level (such as systems and policies). The definition of participation in ICF has been criticised for being vague and only recognising participation in terms of “being there” and excluding the subjective dimension and experiences [7, 8]. Therefore, we include the concept of quality of life defined as psychological wellbeing by Naess [9] which supplements this definition. According to Naess [9] quality of life implies that the individuals’ sense of well-being is sensitive to the influences of life events that are either positive or negative.

Emerging adulthood, a developmental period between the ages of 18 and 29 years [10] is seen as a crucial phase for consolidating identity and for finding one’s place in the social community [11]. Examples of important life situations emerging adults are involved in include independent living, participation in higher education, work, leisure activities and socialising with friends. Emerging adulthood has been identified as a particularly sensitive and challenging time for young individuals living with long-term health challenges [12, 13]. The move away from family influences towards independence and self-determination can cause great uncertainty [10]. Young people with long-term health challenges may concurrently experience changing or declining health and daily symptoms of pain, fatigue and profound physical disabilities, forcing them to depend on equipment and support from care providers to complete daily activities [14].

Previous research has shown that emerging adults with long-term conditions are, in general, less likely to participate in higher education compared to their peers without long-term conditions [15, 16]. The severity of the disease and strong treatment side effects are associated with worse school experiences and outcomes [17]. A Norwegian study showed that 64 per cent of young people between the ages of 25 and 45 years living with a physical disability had not completed secondary education, compared to 17 per cent of the general population [18], while a study from the United States found that fewer than one in five young adults with childhood-onset long-term condition graduated from university [16]. A recent systematic review suggest that students with long-term health challenges must often work beyond their capacity in order to succeed in higher education and access opportunities for meaningful employment [19].

Poor school experiences and outcomes contribute to higher rates of unemployment among emerging adults [20, 21]. Employment fosters the acquirement of life skills and the autonomy needed to participate in society; it also positively influences income levels and increases individuals’ sense of control over their own lives [22]. Previous research has shown that finding and maintaining employment is a major challenge for young people with long-term health challenges, resulting in early work disability [23]. A recent study from Norway documented the negative effects of disability on call-backs from employers on job applications [24]. In their longitudinal survey, Bal et al. [25] found that recipients of disability benefits reported higher perceived impact of the long-term health challenge on their school/career and a lower quality of life than those who had not received disability benefits in young adulthood.

The process of adjusting to and coping with a medical condition can be challenging, inhibiting social functioning [26, 27] and identity exploration and prolonged dependence on caregivers [13]. Due to limitations in their physical abilities and environmental barriers, these young people often have fewer opportunities to engage in recreational and leisure activities [28, 29]. Meaningful contributions to and integration in the community in different areas of life are beneficial and largely contribute to promote personal quality of life [30].

To our knowledge, there is limited research on the experiences of participation in everyday life and quality of life among young people living with long-term health challenges. Previous research has traditionally focused on children or adolescents, there is a limited number of studies on emerging adults. Moreover, previous research on this topic in this population is highly dominated by quantitative research. There is a need for in-depth knowledge to gain a deeper understanding of the experiences of participation in the everyday life of emerging adults living with long-term health challenges and how this influences their own quality of life.

### The Norwegian context

The Norwegian health and social care is based on a welfare model dominated by public funding and the provision of universally available services [31]. The municipalities are responsible for primary health care and the state responsible for specialist healthcare services. The municipalities are responsible for the long-term care services, general practitioners (GP), physiotherapists and emergency care. Specialist healthcare includes both private specialist healthcare providers and hospitals. Residents are assigned a GP who acts as a gatekeeper to the specialist health services. Service delivery, financing, and governance of support for young people living with long-term health challenges are often decentralised to the local level in Norway [32]. Young people living with long-term health challenges may apply for user-controlled personal assistance in the municipality in which they live. User-controlled personal assistance is an individualised, tailor-made service. The users exercise maximum control over how it is organised and custom design their personal assistance service according to their individual preferences, which includes hiring, training and managing personal assistants [33]. To be entitled to assistance in the form of user-controlled personal assistance, the individual must be under 67 years of age and need assistance for more than 32 h per week over a period of more than two years. The legislation furthermore states that individuals who need between 25 and 32 h of personal assistance each week may be entitled “unless the municipality can demonstrate that such an organisation will result in substantially increased costs for the municipality” [34, 35].

## Methods

### Design and data collection

An explorative qualitative design was chosen. The in-depth interviews were conducted between December 2020 and April 2021. Young people living with long-term health challenges were recruited nationwide via specialist healthcare services and non-governmental disability organisations in Norway where this study was conducted. Due to the restrictions caused by the COVID-19 pandemic we planned to conduct the interviews via Zoom. This gave us the opportunity to reach participants of a wider geographical range. This also most likely made it easier for individuals with long-term health challenges to participate in this study. Recruitment methods were written invitations, web advisements and/or oral presentations given by the first author, conducted in collaboration with key healthcare professionals. Inclusion criteria were: [1] young people with a long-term somatic condition or physical impairment which they were born with or which developed at an early age; [2] being aged between 18 and 29 years; and [3] needing accommodation or service assistance due to a long-term health challenge. In this study, 12 young individuals (three men and nine women) with an average age of 23 years (range: 18–29 years) participated and shared their own experiences of living with long-term health challenges. The participants lived in both rural and urban areas. Seven participants lived alone, three participants lived with their parents and two participants lived with their partner. The characteristics of the participants are presented in Table 1.

**Table 1:** Participants characteristics

Participant code	Long-term health challenge	Current main activity
P1 P2	Dystrophia myotonica Osteogenesis Imperfecta	Part time employed Gap year (volunteer work)
P3	Cerebral palsy	Part time employed
P4	Spinal muscular atrophy	Student
P5	Spinal muscular atrophy	Full-time employed
P6	Epilepsy	Student
P7	Epilepsy + fibromyalgi	Full-time employed (sick leave)
P8	Hydrocephalus + epilepsy	Student
P9	Cerebral palsy	Student
P10	Spina bifida	Student
P11	Epilepsy	Part time employed (sick leave)
P12	Osteogenesis Imperfecta	Student

The in-depth individual interviews were conducted using the digital platform Zoom. The interviews were structured as a conversation between the interviewee and the first author (A.R.), to encourage the young adults to speak freely and share their experiences. According to Brinkmann and Kvale [36] in an interview conversation, the researcher asks about, and listens to, what the participants themselves tell about their lived experiences. The interviewer aims to obtain descriptions from the interviewees’ lived world by listening to their dreams, fears, hopes, as well as their views and opinions.

An interview guide was developed consisting of open-ended questions related to everyday life experiences (e.g., possibilities and limitations in their daily life, education and vocation issues and social and societal relations). Two pilot interviews were conducted prior to the interviews included in this study to ensure that the interview guide worked as intended. The pilot interviews were not included in the final data set. There were no significant changes in terms of questions following the pilot interviews. All the interviews started with the opening question ‘Tell me about yourself’, followed by ‘Describe an ordinary day in your life’. The follow-up questions depended on what the young adults talked about. The average length of the interviews in the study was 54 min (range 31–82 min). All interviews were recorded and transcribed verbatim in Norwegian.

### Data analysis

The analyses were inspired by phenomenological and hermeneutic approach for qualitative research [36]. This is an approach that enable us to describe and interpret the subjective experiences of people as they live in the world. Phenomenological research is descriptive and is concerned with the structure of experiences that give form and meaning to the life world. Hermeneutic research is interpretive and focused on meanings of experiences [37]. Thus, phenomenological hermeneutics is concerned with the human experience as it is lived and seeks to elucidate these lived experiences with the goal of creating meaning and reaching a sense of understanding. The purpose of the analytical approach was to extract the meaningful content of the participants’ experiences. Following Brinkmann and Kvale [36] guidelines, the data were analysed in three different contexts of interpretation. In the first step, the self-understanding context, the transcribed interviews were read and re-read several times and summarised to gain an overall understanding. Further they were coded, and categories were assigned using descriptive terminology to capture structures of the participants’ lived experiences. The second step involved a number of structural analyses to grasp the most probable interpretation on meanings of these experiences in the ‘common sense’ context. In this step, the researcher asked questions such as ‘What does this tell us about how they experience their everyday life?’, ‘How does their long-term health challenge influence their home and school/working life?’, ‘How does it influence their ability to engage in leisure and community activities?’, and ‘How does this influence their quality of life?’

This analysis revealed a number of patterns. In the third step, the findings were interpreted and discussed in light of previous research and the theoretical perspectives. The analysis of the text and the synthesis of the findings were mainly performed by the first author. The main themes, illustrative quotations and interpretations were discussed with the co-authors. The co-authors read the Norwegian interviews to validate the first author’s analyses. The researchers have professional backgrounds in the health and social fields and clinical and research experience working with young people with long-term health challenges. We used HyperRESEARCH software [38] to help organise and categorise the transcriptions.

### Ethical considerations

The Regional Committee for Medical and Health Research Ethics for the Southeast Region of Norway determined that the study was not a medical or health-related research project regulated by the law of health research and was therefore not subject to approval. This study was approved by the Norwegian Social Science Data Service (2020/372,502). It adhered to all legal requirements for the protection of personal information. All participants signed a consent form after receiving written and oral information about the purpose of the study. They were informed that they could withdraw from the study at any time without justification and without consequences to themselves. The qualitative interview is an interaction between researcher and the participant; however, the interview entails an asymmetrical power relation. The researcher has scientific competence and the power to determine the interview topics, poses the questions and decides which answers to follow up [36]. The first author conducting the interviews strived to reduce the hierarchy between herself and the participant by creating an emotionally compassionate and supportive interview setting. Signs of discomfort or unease in the participants was monitored carefully. The flexibility of online qualitative methods can be advantageous for marginalised or vulnerable groups, such as young people living with long-term health challenges, who might have difficulty travelling to an in-person interview due to their physical condition or barriers in transport [39, 40]. Due to the online interview setting, the participants were free to choose their own choise of location which might have increased their sense of safety, security and empowerment [40, 41].

### Findings

The interpretation process revealed one overarching theme: experiences of *struggling to participate in everyday life*. In describing their everyday lives, the participants consistently talked about the struggles they faced in participating in different areas of life. They emphasised that everything was much harder for them than it was for others not living with the same health challenges. Everything they did in the course of a day was time consuming and often left them feel exhausted. They described constantly performing ‘a hidden job’ of structuring, planning and prioritising everyday chores and activities. They perceived this process as a prerequisite for them to be able to fully participate in everyday life as they desired.

This overarching theme encompassed four subthemes: the notion of being independent but also dependent, experiencing mismatch between needs and support, experiencing deprivation of spontaneity, and feeling uncertain about the future (Table 2) (See appendix for supporting information).

**Table 2:** Overview of the main findings

Struggling to participate in everyday life			
**Independent but also dependent**	**Mismatch between needs and support**	**Deprivation of spontaneity**	**Future uncertainty**
Striving to be independent	Needs not met or misunderstood	Many obstacles to socialise	Deteriorating health
Overwhelmed by the condition	Struggling with schoolwork	Planning and preparing daily activities	The condition being unpredictable
Coping by showing persistence	Striving to sustain employed	Prioritising necessary activities	Feeling worried about the future
In need of sufficient support	Tired of constant adversity	Feeling included, but on the sideline	Trying to focus on the present

### The notion of being independent but also dependent

The participants described that they found it challenging to manage everyday chores and responsibilities as emerging adults. The intensity of their condition and their capability to manage life on their own varied. Although all the participants strived to do things on their own, many of them were aware that they depended on support, as expressed by one young woman:

"*I’m not sure if I need it, I could probably manage a lot of the things myself. But it really is tough to be a full-time student and also have to deal with everything else in your life. This is actually something that I’m just noticing now, after I’ve left home, all the things that my mum and dad would do to help my life run smoothly. Things like making dinner – they always did that. It meant that I could get on and do the other things that I needed to do – like go to the gym and stuff. Because someone had always made the dinner, and it wasn’t me who had to go out and do the shopping*".

Not all the participants had knowledge of which services and support were available to them.

Some of the emerging adults had full-time support from a personal care assistant to complete everyday tasks. However, not all of the participants had such assistance, and some felt that the help they received was not sufficient. For example, one participant stated:

*"User-controlled personal assistance is an excellent scheme. It’s just very difficult for a lot of people to get onto this scheme, most people don’t even know about it. It’s just that it’s difficult to get the hours you need… You might have the time to take care of your physiological needs, but then you wouldn’t have the time to take care of your social needs. What I think is stupid is that so many people end up in situations where they’re completely dependent on their family and friends"*. 

Being dependent on family members and close ones was not always easy. When the long-term health condition was invisible this was especially difficult. One young woman expressed that she felt that she was not always understood and wished she could ‘take off her mask’ to show everyone why everything was much harder for her. Several of the emerging adults sustained strong-willed and found strategies to manage everyday life despite the difficulties they experienced. For instance, they described preparing and organising for their daily lives by writing down their weekly plans and using phone apps to help them remember their schedules and manage their medications. They talked about how they could be creative about using objects and items in the world in a different way than intended due to their physical limitations. For example, they could use a floor brush to pull something beyond their reach to them if they were alone. One young woman struggling with mild cognitive challenges demonstrated enormous persistence; she explained that she had to maintain a rigid daily routine, often doing the same chores at almost the exact same time every day, to enable her to live independently and ‘survive’ on her own. She had applied for a personal care assistant to help her with daily tasks, but her application was denied on the grounds that she was too “well-functioning’. Being dependent on support when being able to take on certain elements of self-care was an extra hurdle for some of the participants. One young man put it like this:

"*Sometimes I feel that what you might be entitled to doesn’t always take into account those of us who have what you could call a moderate degree of functional impairment*".

### Experiencing mismatch between needs and support

The long-term health challenge and demands of treatment impacted the emerging adults’ school and work experiences. In many ways, they experienced that the support they were offered, both in the school settings and in their workplaces, often did not match their actual needs for help and facilitation. In school, their long-term health challenge influenced their attendance and learning ability, making it more difficult to follow the class curriculum. One young woman explained that this made her feel that she was always falling behind her classmates in different subjects. When she started upper secondary school, she finally got additional help for her reading and writing disability, but she found it frustrating that she had not received this help from the very beginning, when the problems first arose:

"*I think the impact it’s had is that when we start upper secondary school, I haven’t learned the basics the way we should have*".

Some expressed that their teachers lacked understanding of how their long-term health challenge affected their ability to learn. Several participants stated that they struggled with schoolwork due to concentration difficulties, spending hours doing their homework and practising for tests. Others experienced challenges with their schoolwork due to impaired physical abilities. One young man explained his difficulties with writing, especially in subjects such as mathematics, where using a computer was not an option. At school, he had an assistant to help him with writing, but at home he depended on his parents. It was frustrating not to be able to spend as much time working on the subject as he wished because his assistant was only available to him at school. The types of aids offered by schools were a sensitive and significant issue for some of the participants. A sense of helplessness was described by one young man who had failed his exams in one subject twice in his first year at university and was preparing for his third chance to pass. He explained that he needed someone to read assignments for him to ensure that he understood them correctly, but the university he attended did not provide such assistance. Another participant explained how bodily pain and headaches had caused her to postpone her exams several times because the pain was exacerbated when she had to sit in an exam room for several hours. She pointed out that she achieved a better grade on an exam when she had the opportunity to sit at home and work on an exam paper during the COVID-19 pandemic:

"*it’s about not having to be so stressed about it. For me, the coronavirus has actually been a kind of blessing, although obviously I’m sorry that it’s caused so much suffering. Online learning has been better for me than physically going to school*".

The participants also described challenges with work life due to long-term health condition. Pain, fatigue and other associated symptoms of their diagnosis became a part of everyday life to which the participants had become accustomed to a certain degree, but the discomfort still had a negative impact on their occupational performance. Some reported experiencing serious side effects from their medication, which also caused challenges with participation in employment. They were therefore familiar with being on sick leave for short and long periods and working part time; some of them simultaneously received a disability pension. Although they strove to work full time, they found it extremely difficult to do so. One of the participants shared her disappointment:

*“I get really depressed. I get really…. In my head, I’m like everyone else. You’re just being lazy, it’s not the illness. He can do it, she can do it, so you can do it too. And that’s how my mind works”.*

One young woman who realised that she could not push herself constantly to work when her health did not allow it explained that she needed financial support and had applied for a disability pension but had been turned down. Instead, she was assigned to an occupational training programme, supposedly more tailored to meet her individual needs. However, the programme did not go as she expected and left her feeling frustrated and resigned. She elaborated:

"*The try-out has really got in the way of work generally, and it’s made things really difficult for me. Now I’ve more or less given up. I do whatever they tell me to. The hours I am working is actually enough, so that they don’t push me and send me somewhere I don’t feel comfortable, and that’s also bad for my fingers*".

### Experiencing deprivation of spontaneity

‘Hanging out’, socialising and participating in activities with friends were important to all of the participants, but they did not always find it easy. One young woman put it like this:

"*I have a few friends who are always in a rush. It’s like: ‘Shall we do it in 30 minutes?’, ‘Yeah, OK. Fine.’ They don’t think about time in the same way that I do. Because they can just do things whenever they want*".

Spontaneous activities with friends were perceived as challenging for most of the emerging adults in this study. Several of the participants struggled with a lack of energy, exhaustion and bodily pain, which prevented them from seeing their friends as much as they liked to. Many described having to prioritise daily activities. For instance, one participant stated that she was constrained by the amount of physical energy she had: if she had been to work that day, she knew that she would not be able to meet up with friends later on and had to plan accordingly. Another participant explained that she constantly pushed herself to be social with friends, although she knew she would be tired because having fun was also a means of restoring her energy.

Several participants stated that sometimes their physical environment was a reason for them not to ‘take the chance’ to be spontaneous. One young woman recounted an occasion when she was going to attend a Christmas concert at a big church in the city. She was almost certain that she could get in with her wheelchair, but when she arrived, she found that there was only a long staircase and no means of access for those with wheelchairs. Due to that incident and several similar experiences, she stated that she had learned her lesson and accepted the fact that she always had to plan and check beforehand. One of the participants expressed that she often felt that she could not burden her friends with her personal problems, as they already had to adjust to her physical limitations, and everything being prepared and arranged beforehand. Several of the participants reflected on how they wished to feel freer to do whatever they wanted without having to think so much about it. They stated that their acquired limitations sometimes made them feel ‘*included, but on the sideline*’. One young man gave an example of this by sharing an experience with his classmates during a school lunch break:

"*I mean, they’re really inclusive. They’re so nice. We have great chats when we get our ten minute breaks. Then I feel part of the group. We have a bit of a laugh. But I feel that I don’t always get … It’s really hard to find the right words. This thing about being part of this gang at the shop and hanging around in the shopping centre, going back again – it all gets really difficult*".

He elaborated on his desire to join his classmates during their visits to the mall at lunch break. He was prevented from doing so because, unlike his classmates, everything he did was time consuming and often needed a lot of preparation: among other things, he had to change his wheelchair, and he did not have time to do all of that during his lunch break.

### Feeling uncertain about the future

Deteriorating health and physical function pushed life out of balance and consumed feelings of uncertainty for some of the participants. One young woman found herself daily discovering that she could no longer do certain things easily, or in some cases at all, as her health challenge progressed. As she fluctuated between acceptance of the reality of her progressive condition and the experiences of change, the realization of her unpredictable acquired limitations triggered feelings of despair for the future:

"*I notice at work that I can’t manage frozen goods any more. Like I can’t pick up the big box. It really hurts if I try to lift a 20 litre container of oil, when I used to be able to easily stack a whole pallet-load. Things like that. Only being able to work half the hours I used to, then a quarter of the hours. Now my dream job is being advertised, a full-time job that I always wanted, and a position is available. But I can’t apply for it, because I won’t be able to manage it. I’ve had problems with energy for ages, and there are times when you just think: Is this ever going to end? And it doesn’t. I think: How am I supposed to live, sleeping practically all the time, and hardly able to do anything?*"

Another young woman tried to focus on the present in order to keep the uncertainty at bay. She described that she loved her job very much and that she had to work as much as she was doing for the time being and make the most out of it because she knew that eventually she would not be able to work full-time. Adjusting to the unpredictability of the health condition also meant having to make tough decisions that increased uncertainty about the near future, as one young woman expressed:

"*Now I’ve had surgery and the surgery has resulted in more complications than I’d expected. My health is totally different now. I’m pretty tired really. I really just want another year of resting, but I’m not sure, because I don’t really want to fall so horribly far behind all my friends. I just don’t know*".

Another aspect of uncertainty about the future was related to systemic services and support. The participants worried over whether they would be able to get all the support they needed if and as their health challenge progressed, such as a disability pension and increased help from a user-controlled personal care assistant.

## Discussion

This study aimed to gain a deeper understanding of participation and its influence on the quality of life among emerging adults living with long-term health challenges. Our main finding is that the participants in this study struggled to participate in activities of daily living and to manage their responsibilities as emerging adults. From our participants’ accounts, it was clear that they experienced feelings of frustration and hopelessness and that they struggled with concerns about not being able to perform in school and in employment and engage in social activities as they wished and expected to be able to. This clearly influenced their quality of life negatively. The ICF model stresses the dynamic interaction of a person’s health condition and contextual factors affecting the outcome on an individual’s functioning and participation [4]. Many emerging adults experience increased autonomy as they establish independent living and begin to rely less on support from their parents [10]. However, due to the intensity of their health condition and their limited physical capabilities, the emerging adults in this study described fluctuating between trying to transition to independence and still being dependent on care from their parents. Family socioeconomic status may determine the types of involvement and support strategies parents can afford [42]. Swartz et al. [43] found more highly educated parents to be more likely to provide resources. School enrolment, employment problems and negative life events were associated with a greater likelihood of receiving support. Their findings suggest that parents acted as “safety nets” to aid their children’s successful transition to adulthood [43]. The participants in our study recounted the importance of both practical and economic assistance from their parents, as well as support with their schoolwork. Some researchers have expressed concern that parental support in young adulthood prolongs dependence [44, 45]. Others have argued that parental aid helps young people navigate the numerous challenges of early adulthood and supports their capacities for self-sufficiency [46, 47]. For young people living with long-term health challenges the literature indicates that the socially dominant notion of “independence” is not an option, as these young people are often dependent on supports, services and systemic resources [12]. Hence, the notion of interdependence is more appropriate, which suggests a partnership in an effort to maximise one’s potential [48, 49]. Within this perspective independence becomes a two-way responsibility and not solely an individual ability [48]. For the young people in this study however it appeared important that the *partnership* they had with their parents was “replaced” by professionals in the service sectors.

Despite that there is a comprehensive welfare system in Norway, the participants in our study described not receiving the systemic support they needed. Several of the emerging adults in this study exposed feelings of anger and resignation when sharing their stories about repeated negotiations with the Norwegian welfare administration regarding access to a user-controlled personal assistant, how many hours of assistance they should receive and for which purposes. Access to user-controlled personal assistants lie within the competency of relatively autonomous and independent local governments in Norway [32]. According to Brennan et al. [32] local governments fear a rise in the cost of personal assistance, and therefore the guidelines that determine access to personal assistants are often restricted to control the demand. The challenges faced by young people with *moderate* health challenges may therefore be underestimated in the service system. The young people in this study explained how they had been denied sufficient support because they were judged not to be disabled in the right way or disabled to a sufficient extent. There is an experience of power imbalance and unwanted dependency in negotiations with the social services, which has also been found in other studies [50, 51]. Lack of sufficient systemic support made it difficult for the participants to transition to a life independent of parental support and constrained participation in important areas of their life, such as education, employment and social activities. Therefore, the daily activities of the young people in this study appeared to be limited by decisions in the welfare system, thus restricting their opportunities in life to the possibilities provided by welfare services.

Difficulties with school attendance and challenges with schoolwork related to their health condition and its treatment interfered with the participants school performance resulting in feelings of lagging behind in school subjects. In Norway, students living with disabilities are entitled to individual accommodation as long as it does not impose a disproportionate burden on the educational institution [52]. However, participants efforts to access individual accommodation such as flexibility with absences, sufficient support from school assistants in preparation for examination and special examination arrangements was not always successful. Several of the emerging adults described the school staff of having limited knowledge and understanding of their long-term health challenge and their ability to learn in different subjects. Lack of knowledge among educators can create more contextual barriers, such as attitudes that prevent adjustments according to the needs of young people with long-term health challenges [53]. According to Langørgen and Magnus [54] barriers in higher education in a Norwegian context is described to comprise predominantly matters that the individual living with disabilities must resolve on his or her own. When regulatory conditions are based on an individual deficit approach, students’ academic success depends on their individual ability to advocate for their needs and the understanding and willingness of the school staff, and not a result of a formal strategy adopted by the educational institution [54, 55]. Magnus and Tøssebro [56] argued that higher education has long traditions adapted to the image of a typical student, which matches neither the necessity of special accommodation nor the requirements of universal design. The resistance students meet when asking for accommodation is an obstacle to higher education, in spite of legislation and political aims in Norway promoting inclusion [56].

Challenges in sustaining employment among individuals with declining health and functional ability have been reported [23]. Our findings confirm the work of Minis et al. [57], who found that although persons with neuromuscular disease preferred to work instead of having to rely on social benefits, having to work for economic reasons was experienced as a burden, especially when they experienced symptoms which affected their employment attendance and performance. Moreover, certain aspects of the welfare service system presented hindrances to continuing to work as much as they preferred among some of the participants. Experiences of being denied services or being provided inadequate services, e.g. put on unsuitable labour market training programmes. Jaeger et al. [53] argued that, in order to offer the right support and services, professionals need to have syndrome-specific knowledge to balance possibilities in relation to the actual impairment and the person’s expectations and needs. This line of argument was confirmed by the participants in this study.

A sense of unfairness and mourning was present among some of the participants in this study, as their condition could be seen as a potential threat to their freedom to live their lives unconstrained by their condition. This finding has also been reported in previous research [58]. This may involve having symptoms such as pain and, fatigue and living a life that is restricted and controlled by treatment regimens [59]. Several of the participants in this study spoke of their reduced ability to attend leisure activities with friends after school or work, although involvement in such activities was highly valued. Having to focus on juggling the necessary everyday life activities consumed most of their effort and energy, as described in previous work [57]. Furthermore, Melbøe and Ytterhus [60] found that it is not always possible for young people with long-term health challenges to access leisure activities without relevant accommodation and support. Our findings shows that environmental factors such as inaccessible entrances to public buildings limited participants freedom to participate in activities. Barriers in the physical environment can be seen as symbolising the position of people living with disabilities, they are not being prioritised or valued and included as everyone else. Precisely how the community welcomes or discourages public participation is, therefore, an issue of primary concern for enhancing their role as full members of society, with equal opportunities [61].

The findings show that the young people found it especially difficult to participate in everyday life when living with an unpredictable and progressive condition. This created uncertainty and caused them stress, worry and fear for the future [62, 63]. As described in a study by Luft and Koch [64], the young person may resent his or her body, or even fear it at times, when the body as the young person used to know it has changed. According to Imms et al. [65], young people choose what they will participate in based on prior participation experiences and expectations of/for future participation. Awareness of the loss of physical function and declining health has been found to make it difficult to pursue personal goals [63, 66].

### Strengths and limitations

This study is limited to the experiences of a small group of young people with long-term health challenges. The individuals we recruited were probably among the most outgoing and resourceful members of the target population, as several of them contacted the research group themselves and were willing to participate in the study after seeing web advisements about it. Other participants were recruited from disability associated organisations. More vulnerable individuals are less likely to take the initiative to participate in research or engage in disability associated organisations. Nevertheless, the individuals who participated put their feelings and experiences into words, and we believe these may be transferable to many emerging adults living with long-term health challenges.

The digital tool Zoom was used to conduct the in-depth interviews for this study due to the restrictions caused by the COVID-19 pandemic. One of the participants experienced technical problems (a malfunctioning camera), and therefore nonverbal expressions were not captured by the researcher during that particular interview. This may have resulted in lack of asking relevant follow-up questions and, consequently, a loss of valuable information. Nevertheless, the participants in this study provided rich narratives relevant to the phenomenon under exploration. The trustworthiness of the findings has been increased by providing accurate quotations from the young people in this study, thereby enabling their first-person experiences to be acknowledged and given voice. Finally, the discussions within the research group during the analysis process provided important reflections and interpretations about the descriptions given by the emerging adults regarding their experiences of living with a long-term health challenge.

## Conclusion

Living with a long-term health challenge as an emerging adult affected participation in everyday life and quality of life in various ways. To participate in important areas of life was perceived as crucial for their experience of quality of life. The emerging adults experienced distress, worry and felt sad about the restrictions imposed on them by living with a long-term health challenge. They found living with pain, fatigue and physical limitations exhausting. It was challenging to participate in different areas of life when everything needed to be planned and prepared. The lack of sufficient systemic support increased their difficulties in participating in everyday life, which again made it difficult to gain independence and rely less on parents to lead a full life. To enhance participation opportunities and quality of life for young people living with long-term health challenges, the mismatch between their individual needs and the support they receive must be addressed. Knowledge and understanding among professionals working in the health, educational and social services sectors is essential in order to enable equal participation opportunities for all young people, regardless of their disabilities.

## Electronic supplementary material

Below is the link to the electronic supplementary material.

Appendix: Table 1 Overview of the main findings

## Data Availability

The datasets analysed during the current study are not publicly available due to privacy/ethical restrictions but, are available from the corresponding author on reasonable request and with permission from the Norwegian Social Science Data Service.
