# Supplementary material for: ‘Struggling to participate in everyday life’: emerging adults’ experiences of living with long-term health challenges
Source: BMC Public Health. 2023 Jul 17;23:1368. doi: 10.1186/s12889-023-16291-6 (PMC10353226; doi:10.1186/s12889-023-16291-6)
Supplement: Supplementary file 1 — Appendix: Table 1 Overview of the main findings [file 12889_2023_16291_MOESM1_ESM.docx]

**Appendix**

Table 1 Overview of the main findings

| **Struggling to participate in everyday life** | | | |
| --- | --- | --- | --- |
| **Independent but also dependent** | **Mismatch between needs and support** | **Deprivation of spontaneity** | **Future uncertainty** |
| Striving to be independent  To me, my independence is very important. It gives me a sense of joy to do things on my own. It’s not about being as good as or better than someone else. It’s purely because I want to manage things on my own. It’s a battle everyday, because not many things are adapted in today’s world. | Needs not met or misunderstood.  For example, when a lady from the municipality visited me. I was going to apply for UPA. She asked so many questions, well, not actually a lot of questions, she was more guiding than anything. “You have a microwave, don’t you? “You can make ready-made meals.” I stuttered, “Yes, but I also want nice healthy meals, like you can make. I don't just want to settle with for example ready made meat balls, when I can actually make lasagne or meat balls from scratch. You’re not respected or understood. | Many obstacles to socialize  With the additional problems, like no adaptation ... things are different for me. Let’s say that I want to get the bus somewhere. It’s not guaranteed that the ramp will work. Then I have to wait another thirty minutes and stand outside longer. The bus driver might not even bother picking me up because he can’t be bothered to put the ramp out. Then I have to wait another hour for the next bus. There are many things like that. | Deteriorating health  So, if the disease doesn’t stagnate soon, the thought of deteriorating health is actually very daunting, because it happens quickly. I haven’t noticed it much, as it stagnated when I was 14 up to the age of 18. Now, I discover one thing every day that I can’t do or have problems with. |
| Overwhelmed by the health condition  I didn’t have assistance for around nine months, which was actually very hard and difficult. It was so exhausting that I was always fainting all over the place. I was so tired when I got home that I just had to lay down. Wherever I was, I just laid down and went to sleep. | Struggling with schoolwork  I have to constantly explain and say what’s wrong, but they ... Just like ... We have a drug dosage calculation exam. It hasn’t gone well a couple of times. It’s my third attempt now. I’d like someone to read the questions for me. According to the guidelines, it’s allowed. But they say it isn’t. I’m 19 years old and a new student, so what can I say against school management about that? | Planning and preparing daily activities  Normally I have to create a plan on which I’ll write: tomorrow I’ll spend my day travelling and do some grocery shopping or I’ll spend the day visiting my parents. I feel like I have to ... It’s the same when I’m at work. It’s good to know when I have to go to work, at least, two days ahead, so I know that I’m going to work that day and won’t be doing anything else. | The health condition being unpredictable  They appear randomly [symptoms of the condition]. Sometimes they appear every day for three weeks, then they disappear for a month or just one week or three days, and then they appear again. They fluctuate a lot. |
| Coping by showing persistence  I’ll have my health condition my whole life. I just have to choose whether to accept it or not. I’ve accepted it and therefore I have to have many routines. They get me through the day, so I don’t forget things, and turn up at work on time and so on and so forth. | Striving to sustain employed  I can have an extra five minutes at break times if I need it. Other than that, I don’t have any special measures or granted rights at the present time. I’m thinking about applying for UPA again or complaining about the last outcome. I’ll then get something called a functional assistant. The person will at least be with me at work. Besides the extra five minutes, I don’t get any help or adaptation. | Prioritizing necessary activities  When you don’t get the assistance or help that you need, and when nothing is adapted, very often it’s just a matter of surviving. You go to work because you have to go to work. If you want to go to a party afterwards, you don’t have the energy or resources to go because you’ve spent all your energy on doing what you had to do. | Feeling worried about the future  I have 50 hours now [UPA]. I have 10 hours every day from Monday to Friday, but what will it be like when I leave home? Will I have to have a community nurse at nights because I don’t have enough hours for 24 hours with UPA. |
| In need of sufficient support  I need help with most things, and if I get it, I will be quite independent and capable of, for example, working or getting an education and living alone. | Tired of constant adversity  The application process has been difficult, applying for a car or assistive devices and having to argue to get them. Saying, “Yes, I still have a disability.” In some ways the system is not always adapted to adapt. At least when you’re in the system your whole life not just a short time. | Feeling included, but on the sideline  Even though I might think it’s difficult being in a wheelchair sometimes, they’ve still been there [friends]…Speaking of not wanting to take up so much space, give such a big impression of what I need…there is something left unsaid there. Which, I’ve experienced many times. Maybe it's the insecurity rather than them being directly embarrassed, but I connect it to something negative, which perhaps makes me a burden. | Trying to focus on the present  After all, it’s a progressive disease and I don’t know how quickly it will get worse. The uncertainty affects me quite a lot in periods. I have a hypothesis that I must work as hard as I do because I don’t know when I won’t be able to work as much. |

Note: User-controlled personal assistance (UPA). The citations are from minimum 7 participants.
